# A modification with threading cannula needle-assisted 4-point suspension fixation for retroperitoneal laparoscopic pyeloplasty in children with ureteropelvic junction obstruction: a cohort study in single center

**DOI:** 10.1007/s11255-018-2048-x

**Published:** 2018-12-05

**Authors:** Ke Li, Cheng Hu, Wentao Huang, Jie Si-Tu, Li Lu, Yunhua Mao, Huimin Zhang, Jianguang Qiu, Dejuan Wang

**Affiliations:** 1 Department of Urology, The Third Affiliated Hospital of Sun Yat-sen University, Tianhe Road 600, Guangzhou, 510630 Guangdong China; 2 Department of Urology, The Sixth Affiliated Hospital of Sun Yat-sen University, Yuancun Erheng Road 26, Guangzhou, 510655 Guangdong China

**Keywords:** Children, Ureteropelvic junction obstruction, Retroperitoneal laparoscopic dismembered Pyeloplasty, Suspension fixation, Anastomosis

## Abstract

**Purpose:**

To evaluate the effect and safety of modifying a threading cannula needle-assisted suspension fixation in retroperitoneal laparoscopic dismembered pyeloplasty (LDP) for children with congenital ureteropelvic junction obstruction (UPJO).

**Methods:**

Between December 2012 and December 2017, 45 children (< 14 years of age) with congenital UPJO were divided into two groups. In Group A, children underwent conventional “no-suspension fixation” LDP; and in Group B, “4-point suspension fixation” LDP was performed to lower difficulties and shorten operative time. The perioperative clinical data were recorded and analyzed.

**Results:**

No statistical difference was found between two groups in preoperative characteristics. The duration of surgery, operative time for completion of anastomosis and the length of postoperative hospital stay in Group B was remarkably shortened than that in Group A, respectively (*P* < 0.05 for all). There was no significant difference between two groups in terms of postoperative renal pelvic diameter (RPD) decreasing, extubation time and success rates of surgery (*P* > 0.05 for all). In addition, no recurrent stenosis and urine leakage in both groups, and the postoperative RPD remained at the low level in both groups during the period of follow-up.

**Conclusions:**

Our modification of the 4-point suspension fixation for retroperitoneal LDP is an effective and safe method for children with UPJO. It can simplify the surgical procedures, lower difficulties (especially in precise anastomotic suturing) and shorten the learning curve. This modification might be of particular interest to urologists for improving treatment of children with UPJO.

## Introduction

The prevalence of ureteropelvic junction obstruction (UPJO) in children is about 1/200,000, 25% of whom require surgical intervention. The gold standard surgical treatment is Anderson–Hynes pyeloplasty [1]. At present, open pyeloplasty (OP) is still the mainstream for children with UPJO, but pediatric robot-assisted and laparoscopic dismembered pyeloplasty (LDP) is being widely used [2]. A vast number of studies have shown that the efficacy and safety of LDP is comparable to that of OP. In addition, the laparoscopic approach is associated with less tissue injury and less pain, shorter hospital stay, as well as a cosmetic incision [3–5].

However, since the operable space in a children’s body is small for laparoscopy, performing precise anastomotic suturing is restricted and the duration of operation is longer than OP, especially in LDP by retroperitoneal approach [6, 7]. Furthermore, it is easy to cause accidental damage to the surrounding tissues, resulting in bleeding and an increased risk for anastomotic stenosis for LDP in children [8]. The laparoscopic operation is more challenging and requires a longer learning period for urologists [9].

In the present study, we introduce the modification that improves LDP surgical procedures by retroperitoneal approach using a threading cannula needle-assisted 4-point suspension fixation in treatment of children with UPJO < 14 years of age.

## Methods

### Study population and design

The records of 45 children (< 14 years of age) with congenital UPJO who underwent retroperitoneal LDP between December 2012 and December 2017 were retrospectively reviewed. All children received preoperative radiological imagining, including ultrasonography, or computed tomography urography (CTU), or dual renal emission CT (ECT), for the diagnosis of UPJO. The indications of surgery were children with hydronephrosis reaching the Society for Fetal Urology universal criteria (SFU) grade III–IV, recurrent febrile urinary tract infections (UTIs), CTU showed an obvious obstruction, and a radionuclide symmetrical differential renal function of 40% or less. Children who had a previous abdominal and renal surgery, an extremely large renal pelvis (i.e., pelvis diameter > 6 cm), pelvic kidney, and horseshoe kidney were excluded from the study. The study was approved by the institutional review board and the ethics committee of the Third Affiliated Hospital of Sun Yat-sen University, and all research was conducted in accordance with the Declaration of Helsinki. Written informed consent was obtained from all parents.

Retrospective chart review of records of all children was divided into two groups: Group A, underwent conventional retroperitoneal LDP without suspension fixation; and Group B, underwent retroperitoneal LDP using 4-point suspension fixation method. Pre- and postoperative examination data were recorded. The duration of surgery, complete anastomosis time, time to removal of the double-J tube postoperatively, length of hospitalization and any postoperative complications were documented. The primary endpoint was operative time for completion of anastomosis; secondary endpoints were duration of surgery and length of hospitalization.

### Surgical procedures and postoperative management

One surgeon who had experience with a minimum of 50 LDP operations prior to the study period, performed all surgeries.

Before surgery, a disposable cannula piercing needle (18 G, 45 mm; B. Braun Medical Inc., Germany) was threaded with folded 2-0 Tee polymer pledget (Ethibond excel, Ethicon Endo-Surgery Inc., USA). Operations were performed with conventional tracheal intubation under general anesthesia, and children were placed in a lateral position, with the waist elevated.

**Fig. 1:**
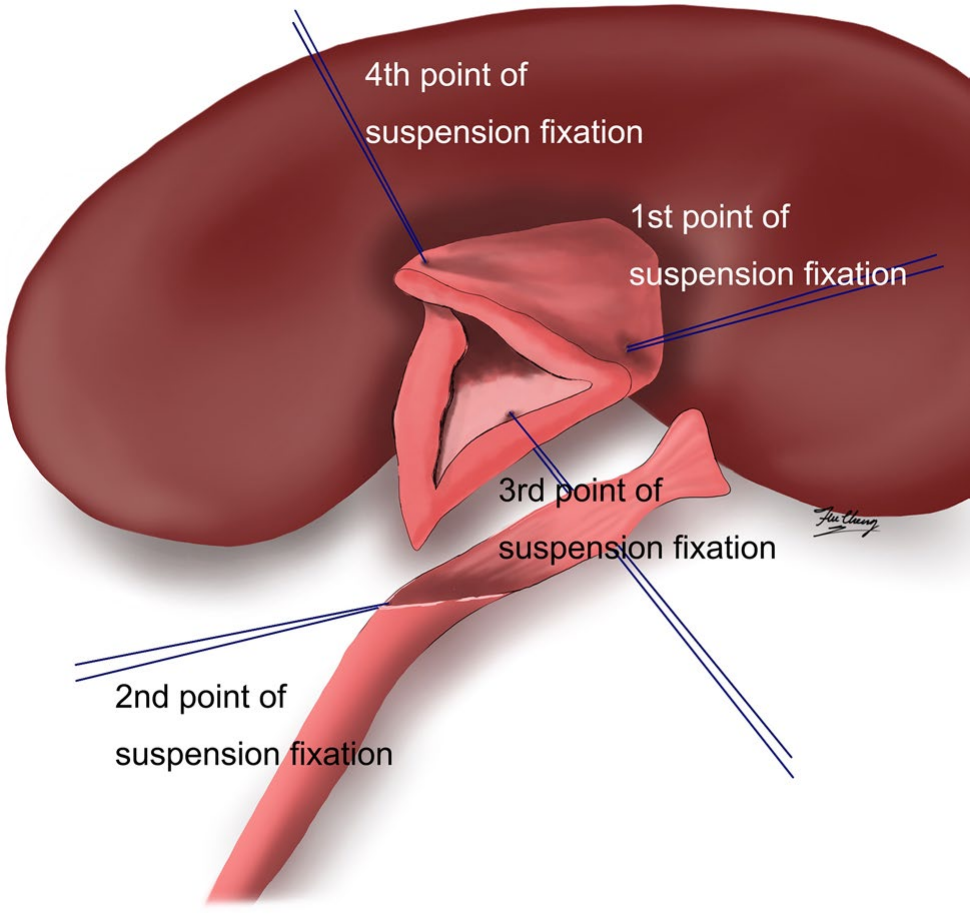
Schematic diagram of assisted suspension fixation in retroperitoneal laparoscopic pyeloplasty on children

**Fig. 2:**
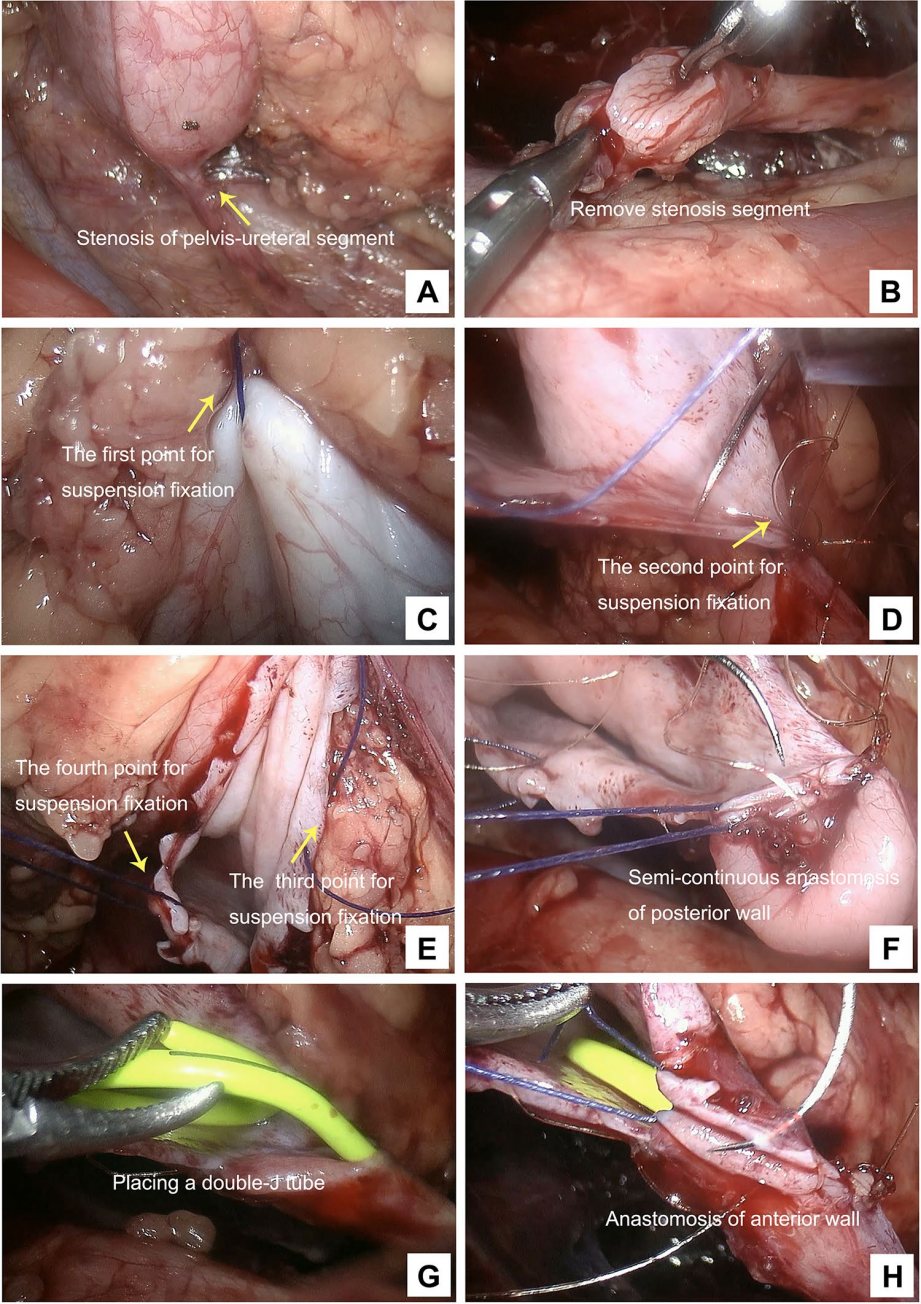
Intraoperative images. **a** The stenosis of pelvis-ureteral segment. **b** The removal of stenosis segment. **c** Suspension fixation at the lateral middle renal pelvis near the highest point. **d** Suspension fixation at the lowest point of ureter. **e** Suspension fixation at the lowest point of renal pelvis and at the posterior wall of the anastomotic renal pelvic incision, respectively. **f** Semi-continuous anastomosis between the renal pelvis and the posterior wall. **g** The placement of double-J tube. **h** Semi-continuous anastomosis for the anterior wall

All patients received the retroperitoneal approach, an incision was made at 2 cm above the anterior superior iliac spine of the affected side to establish a retroperitoneal operation space. A 5 mm trocar and a 3 mm trocar were placed on the anterior axillary line and posterior axillary line, respectively. A 10 mm trocar was placed in the incision above the upper anterior superior iliac spine to establish a retroperitoneal pneumoperitoneum with a pressure of 8–10 mm Hg. For Group A, all children underwent conventional LDP as previous study [10]; while in Group B, 4-point suspension fixation method was fixed (Fig. 1). In detail, the stenosed pelvic-ureteral segment was dissected and exposed, followed by dissociating the renal pelvis to identify the lowest and highest point of renal pelvis (Fig. 2a, b). Next, 4-0 vicryl was used to place one suture at the lateral middle renal pelvis near the highest point with a long line tail.

A threading cannula needle was punctured into the abdominal cavity at the position of the surface projection of renal pelvis under laparoscopic direct vision. The #0 Mersilk in the cannula needle was loosened to form a loop. The 4-0 vicryl line tail at the previous suture was placed into the loop using a forcep. The line tail was taken out of the surface projection of the renal pelvis using the threading cannula needle, and then the line tail was pulled and tightened outside the body (this site was the first point of suspension fixation, Fig. 2c). An everting suture was placed at the lowest point of ureter and the lowest point of renal pelvis and tied with a sufficiently long tail, respectively. The tail of the suture was taken out of the body using the threading needle method, and the site where the line exited the body was near the trocar placed at the anterior axillary line. The suspension line tail was tightened, and was fixed with forcep outside the body (the second and the third point of suspension fixation, Fig. 2d, e). The fourth point of suspension fixation was at the posterior wall of the anastomotic renal pelvic incision, and the posterior wall was pulled by the suspension suture to the right side. The tail of the suspension suture was pulled out of the body above the trocar placed at the posterior axillary line using the threading needle method (Fig. 2e). After the 4 points were suspended, the anastomosis site was fixed by a 4-point fixation method. And then, a semi-continuous anastomosis was performed between the renal pelvis and the posterior wall of the broken end of ureteropelvic segment (Fig. 2f). After placing a double-J tube, a semi-continuous anastomosis was then performed for the anterior wall of the anastomotic site (Fig. 2g, h). Finally, a retroperitoneal drainage tube was placed at the suture site after confirming no urine leakage.

Anti-inflammatory therapy was administered for 2–3 days. The catheter was removed at 2 days after the operation, and the drainage tubes were removed at 3 days after the operation. All patients returned to the hospital at about 1 month post-operatively for double J-tube removal. At 1, 3, 6, and 12 months after surgery, ultrasonic examination was performed to measure the renal pelvic diameter (RPD), and routine urine examination was also performed.

### Statistical analysis

The data were analyzed using the Statistical Package for the Social Sciences version 17.0 (SPSS Inc., Chicago, IL.). The findings were compared using Student’s *t* test, and the incidence of postoperative adverse events was compared using the Chi square test. A *P* value of < 0.05 was regarded as significant.

## Results

### Baseline patient characteristics

A total of 45 children were evaluated. According to the SFU criteria, 34 children had grade IV hydronephrosis and 11 children had grade III. Four children in each group received percutaneous nephrostomy due to recurrent fevers and UTIs before surgery. There were no statistically significant differences between the groups in terms of age, weight, RPD and SFU grade (*P* > 0.05 for all). The baseline characteristics of the study children are summarized in Table 1.

**Table 1:** Baseline patient characteristics in two groups

Variable	Group A (*n* = 23)	Group B (*n* = 22)	*P* value
Gender			
Male	17	15	0.58
Female	6	7	
Age (years)	9.6 (7.5–13.2)	8.8 (7–13.5)	0.85
Weight (kg)	32 (18–41)	31 (19–40)	0.95
Side of obstruction			
Left	16	17	0.56
Right	7	5	
SFU grade			
Grade III	18	16	0.67
Grade IV	5	6	
Preoperative RPD (cm)	3.3 ± 0.7	3.5 ± 0.6	0.34
Indications for surgery			
UTIs	11	7	–
Pain	17	13	
Postnatal hydronephrosis	3	3	
Hematuria	3	2	

*SFU* Society for Fetal Urology, *RPD* renal pelvic diameter, *UTIs* urinary tract infections

### Perioperative outcomes

All children underwent successful surgery. There was a significant difference in the overall operation time between the Group A and the Group B (225.5 ± 30.2 min vs. 196.3 ± 31.4 min, *P* < 0.05). Similarly, the complete anastomosis suture time of Group B was remarkably less than that of the Group A (88.1 ± 16.6 min vs. 130.2 ± 20.5 min, *P* < 0.05). There were no statistical differences in postoperative extubation time between the two groups (*P* > 0.05). In addition, the length of postoperative hospital stay in Group A was longer than that in Group B (4.2 ± 1.3 days vs. 3.3 ± 1.4 days, *P* < 0.05). No major complications were observed during the perioperative period and no children required blood transfusion in both groups. The surgery-related parameters are shown in Table 2.

**Table 2:** Surgery-related parameters in the two groups

Variables	Group A (*n* = 23)	Group B (*n* = 22)	*P* value
Duration of surgery (min)	225.5 ± 30.2	196.3 ± 31.4	< 0.001*
Operative time for completion of anastomosis (min)	130.2 ± 20.5	88.1 ± 16.6	< 0.001*
Postoperative hospital stay (days)	4.2 ± 1.3	3.3 ± 1.4	0.025*
Postoperative RPD (cm)	1.7 ± 0.6	1.6 ± 0.5	0.675
Postoperative days to removal of the double J tube (days)	28.7 ± 7.5	30.2 ± 6.8	0.23

Data are expressed as means ± standard deviation

*RPD* renal pelvic diameter

*Significant

For one child in Group B, it was difficult to place the double-J tube to the distal end, and when performing retrograde placement of the tube, ureteral bladder entrance stenosis could be observed on the diseased side. In this case, percutaneous nephrolithotomy was performed, and the anastomotic soluble hollow stent was removed through the nephrostomy tube. The nephrostomy tube was removed at 10 days after surgery. Otherwise, one child in Group A, and two children in Group B developed post-operative UTIs.

The median period of follow-up for patients in both groups was 12 months (range, 9–65 months). The results showed that no recurrent stenosis and urine leakage in both groups. The RPD was measured by ultrasonic decreased from 3.3 ± 0.7 cm in Group A and 3.5 ± 0.6 cm in Group B to 1.7 ± 0.6 cm and 1.6 ± 0.5 cm, respectively, and remained at this low level during the period of follow-up. However, there were no statistical differences in the RPD decreasing between the groups (*P* > 0.05). The success of operation was defined as improved drainage, as assessed by ultrasound or renal scintigraphy, and the absence of UTIs. When persistence of symptoms with obstruction was demonstrated on functional imaging or subsequent treatment was required postoperatively, the operation was considered to have failed. Overall, the success rate of surgery was 91.3% (21/23) in the Group A and 95.5% (21/22) in the Group B.

## Discussion

Surgical intervention for pediatric UPJO might resolve without any treatment, but there is no method to determine which cases will resolve and which will not [11]. It is also unclear how long the condition should be observed before surgery is indicated, what the effect of early vs. late surgery is on renal function, and how renal dysfunction should be defined. Moreover, the impact of anesthesia on children is uncertain [12]. When it is necessary, treatments such as nephrostomy could be performed in children to preserve renal function so that long-term corrective surgery can be performed at an appropriate age. However, giant hydronephrosis or recurrent urinary tract infection will cause irreversible renal function insufficiency. In addition, there are difficulties in the nursing of nephrostomy tube in children and urinary tract infection can easily occur, therefore, some surgeons chose aggressive surgeries during neonatal period. If surgery was not selected, but after neonatal period, the surgeons can choose Anderson–Hynes pyeloplasty after adequately communicating with the parents in the following cases: (1) the children with UPJO developed grade SFU III–IV hydronephrosis, recurrent UTIs or fevers; (2) CTU showed obvious UPJ obstruction; (3) renal function examination with nuclide showed split renal function of less than 40% [13]. Based on the aforementioned considerations, we use strict criteria for surgical intervention for all children.

There are many surgical methods to treat the condition, among which Anderson–Hynes pyeloplasty has a cure rate of 90–100%, and is considered the gold standard method of treatment [1]. With the development of advanced laparoscopic techniques, experienced centers have carried out laparoscopic Anderson–Hynes pyeloplasty with an efficacy close to that of open surgery and the advantages of minimally invasive surgery. Compared to retroperitoneal approach, there are much more studies that report LDP has underwent the transperitoneal approach in children [14, 15]. The operable space of the transperitoneal approach is relatively large, instruments can be easily inserted into peritoneal cavity and removed, and it is also easier to perform intraoperative suturing [16]. Although the challenge of manipulation certainly increase in retroperitoneal approach, it allows more direct access to the site of obstruction, the incident of intraoperative bowel-related complications and postoperative pain are lower as compared to the retroperitoneal approach. Furthermore, if urine leakage occurs, it will accumulate in the retroperitoneum which is helpful for drainage as abdominal organs are avoided. In addition, it is still possible to perform a second surgery by the transperitoneal approach after the retroperitoneal procedure is unsuccessful [17–19].

We previously introduced the use of threading cannula needle-assisted suspension fixation in laparoscopic surgery to treat hernia or hydrocele [20]. Now we refer the above method and experience for solving the difficulties of laparoscopic anastomosis in children by retroperitoneal approach. This method effectively solves problems such as a small operable space in children and difficultly suturing, and the anastomosis time can be remarkably shortened. In this study, the anastomosis time in suspension fixation group was shorter than that in the conventional suture group, and the difference was statistically significant.

The positions of the 4 points of suspension fixation were as follows: suspension from the middle of the renal pelvis near the highest point toward directly up to the outside of abdominal wall; suspension from the first suture at the lowest point of the anastomotic renal pelvis and the lowest point of the ureteral incision to the left and outside of the abdominal wall; the suture line at the middle of anastomosis posterior wall of the renal pelvis segment toward the right to the outside of the abdominal wall. This method is helpful for intraoperative anastomotic fixation and suture, which can reduce the difficulties of suture in children’s body, precisely judge the entry point and exist point for trocar and solve the effect of short breath of children under anesthesia during operation on operation field, and thus to avoid hands tremble and shorten the time of suture. It is also helpful for exposure, avoiding postoperative edema due to recurrent clamping of anastomotic tissues and thus promoting recovery. In addition, the time of placing double-J tube can be significantly shortened, and thus greatly reducing the learning curve. Moreover, threading cannula needle-assisted suspension fixation is simple, and there is less tissue injury. The tightness of the suspension lines can be adjusted at any time during the operation, and the direction of exposure can also be adjusted for further reducing the difficulty of surgery. For these reasons, our data show that the length of postoperative hospital stay in Group B was also shorter than that in the Group A. Furthermore, if it is necessary, the positions of the threading cannula needle-assisted suspension fixation and the number of needles can be increased at any time during the operation.

This study is limited by its retrospective and observational design. The long-term treatment efficacy still requires investigation. A further study with a larger study population with the well-balanced baseline parameters, which could possibly reduce bias should be conducted to verify our findings.

In sum, our study suggests using a threading cannula needle-assisted suspension fixation to perform LDP by retroperitoneal approach, which can significantly reduce the operation time and surgical difficulties in children, as well as address limitations such as a small operable space in children, allows precise laparoscopic suturing. We expect that these modifications will be applied for learning LDP easily to urologists.
